# A novel method for pair-matching using three-dimensional digital models of bone: mesh-to-mesh value comparison

**DOI:** 10.1007/s00414-016-1334-3

**Published:** 2016-03-10

**Authors:** Mara A. Karell, Helen K. Langstaff, Demetrios J. Halazonetis, Caterina Minghetti, Mélanie Frelat, Elena F. Kranioti

**Affiliations:** 1 Edinburgh Unit of Forensic Anthropology, Edinburgh, UK; 2 School of History, Classics and Archaeology, University of Edinburgh, Edinburgh, UK; 3 Department of Orthodontics, School of Dentistry, National and Kapodistrian University of Athens, Athens, Greece; 4 Department of Cultural Heritage, University of Bologna, Ravenna, Italy; 5 UMR 7268 ADES, Aix-Marseille Université/EFS/CNRS, Marseille, France; 6 Evolutionary Studies Institute, University of the Witwatersrand, Johannesburg, South Africa

**Keywords:** Forensic anthropology, Pair-matching, Commingled remains, 3D geometry, 3D modeling, Mesh-to-mesh value comparison

## Abstract

The commingling of human remains often hinders forensic/physical anthropologists during the identification process, as there are limited methods to accurately sort these remains. This study investigates a new method for pair-matching, a common individualization technique, which uses digital three-dimensional models of bone: mesh-to-mesh value comparison (MVC). The MVC method digitally compares the entire three-dimensional geometry of two bones at once to produce a single value to indicate their similarity. Two different versions of this method, one manual and the other automated, were created and then tested for how well they accurately pair-matched humeri. Each version was assessed using sensitivity and specificity. The manual mesh-to-mesh value comparison method was 100 % sensitive and 100 % specific. The automated mesh-to-mesh value comparison method was 95 % sensitive and 60 % specific. Our results indicate that the mesh-to-mesh value comparison method overall is a powerful new tool for accurately pair-matching commingled skeletal elements, although the automated version still needs improvement.

**Electronic supplementary material:**

The online version of this article (doi:10.1007/s00414-016-1334-3) contains supplementary material, which is available to authorized users.

## Introduction

In forensic anthropology, pair-matching skeletal elements is a common individualization technique for situations involving commingled remains, such as mass graves, or mass tragedies due to natural disasters. Currently, techniques range from visual pair-matching, which is a subjective technique based on the similarities in morphology of left and right paired elements [1], to more objective methods such as osteometric comparison, which uses statistical models and quantitative approaches to compare the size and morphology between elements, paired or not [1–3]. More recently, a geometric morphometric approach has also been applied to the task [4].

All of these methods’ accuracy rates, however, vary considerably. In the only two studies conducted on the method, visual pair-matching ranges from being roughly 75 to 90 % accurate, depending of the type of skeletal element being matched [4, 5]. A geometric morphometric approach seems to be more reliable, at 100 % accurate, but this is only the case if all true pair matches are actually present in the sample population [4]. Otherwise, this method will lead to erroneous results [4]. Furthermore, to our knowledge, there has only been one study conducted on the topic, focused only on metacarpals [4]. Osteometric comparison methods have in some ways side-stepped the problem of matching paired elements by focusing instead on using a null hypothesis which is based on the principle of exclusion [6]. Thus, the accuracy of excluding that two skeletal elements match is predicted using either 90th and 95th percentiles or a 90 % prediction interval, but again appears to be variable in terms of reliability when applied to different skeletal populations [6–8].

The fact that these methods’ accuracy rates vary considerably is not surprising; the accuracy of visual pair-matching often depends on the experience of the osteologist, while both visual pair-matching and osteometric comparison methods acknowledge that anatomical similarity between individuals often complicates individualization. Furthermore, despite the knowledge that paired skeletal elements from the same individual are often quite *asymmetrical*, currently only the geometric morphometric approach acknowledges the effects of bilateral asymmetry with regard to pair-matching [4, 9, 10]. Additionally, as the geometric morphometric approach relies on the consistent landmarking of variable anatomical features, there is always the complication of ensuring that each practitioner can reliably identify and place the required anatomical landmarks, without introducing error [11].

Beyond this variability in the accuracy of pair-matching, it is also important to note the recent push in forensic/physical anthropology to make all individualization methods more objective and reliable, with known error rates [12]. This push paired with the increased use of technology, such as CT and 3D surface scanners, throughout the field of forensic anthropology [13–15] suggests that new technologies could be used to help improve current individualization techniques such as pair-matching, creating more objective and reliable methods, with known error rates.

In this vein, we propose a new method of pair-matching, mesh-to-mesh value comparison (MVC), which directly compares the entire digital three-dimensional morphology of two bones without the need for biological landmarking. This method utilizes a “mesh-to-mesh value,” a single value which quantifies the difference of two meshes (models) in millimeters, though it is not necessarily a simple average of the distances between the two models. Still, the lower a mesh-to-mesh value, the more similar two models, or meshes are. The algorithms used to determine a mesh-to-mesh value are based on Iterative Closest Point (ICP) comparison algorithms [16]. For the initial manual MVC method, using LMI Technologies Flexscan 3D software, other, unknown, proprietary algorithms are also involved in mesh-to-mesh value creation. To account for this, we designed an automated MVC method using an add-on for the software program Viewbox 4 (dHAL Software, Kifissia, Greece), in which the algorithms used to compare mesh similarity could be controlled and the process sped up.

In addition to testing the MVC method, sensitivity and specificity rates were assessed as an alternative analysis tool for accuracy. Though this method of analysis has previously only been used in forensic science for the comparison of diagnostic tests such as the presence of drugs [17] and human saliva [18], it was chosen for this study because it offers more information than simply counting accurate pair matches alone. Instead, it demonstrates the accuracy of a method by giving the precise rates of a method choosing, for example, false pair matches verses true pair matches or true negatives. In this way, pair-matching methods, regardless of the population size, number of pairs, and number of single bones, can be easily cross-compared and understood. Additionally, though current methods generally report type I errors, sensitivity and specificity rates express both type I and type II errors, the latter of which is an underused statistic in biological anthropology [19]. Sensitivity and specificity rates also provide greater information about *how* a method may have misidentified some pairs, elucidating if it was mismatching bones or if it simply did not find a match for a bone at all.

The aim of this study was to determine if digital three-dimensional models of bones could be used to pair-match skeletal elements with a high degree of statistical accuracy, as measured by sensitivity and specificity. This was tested utilizing two different versions of the MVC method, one manual and the other automated. Paired humeri were chosen as a test population on the reasoning that if a method can accurately sort highly asymmetrical unmatched humeri into their proper pairs, it can aid in sorting all paired bones. In this manner, the study acknowledged bilateral asymmetry and its possible effect on pair-matching.

## Materials and methods

### Materials

A total of 45 well-preserved humeri from three different populations (G1, G2, and G3) were used for this study, to insure coverage of multiple time periods and geographical locations. Thirty-one humeri were scanned via computed tomography (CT) and 14 humeri were three-dimensional surface scanned. The main difference between the CT and 3D surface scan data was that the 3D surface data does not include the internal structures of the objects which were scanned [20].

Ten known pairs of humeri (G1) originated from the Ballumbie and St. Andrews medieval Scottish collections (fifteenth to seventeenth century) held by the University of Edinburgh [21, 22]. Eleven humeri (G2), including four pairs and three individual humeri, originated from the archaeological Ibizan cathedral collection (thirteenth to early nineteenth century) in Spain, held by the Ibizan city hall [23]. Seven known pairs of humeri (G3) originated from the Frassetto Collection (Collezione Frassetto) in Italy, a modern collection held by the Anthropology Museum at the University of Bologna. All of these individuals originated from Sassai (Sardinia), died in the first decade of the twentieth century, and were donated to the collection. For a full list of specimens, see Table 1.

**Table 1:** List of specimens including sex, age, and completeness

ID number	Randomized number	Collection	Age	Sex	Completeness^a^
StA-17R	8	Scotland	Adult	–	1
StA-17 L	4	Scotland	Adult	–	1
StA-26R	1	Scotland	Adult	–	1
StA-26 L	14	Scotland	Adult	–	1
StA-35R	17	Scotland	Adult	–	1
StA-35 L	19	Scotland	Adult	–	1
StA-36R	25	Scotland	Adult	F	1
StA-36 L	30	Scotland	Adult	F	1
StA-47R	34	Scotland	Adult	–	1
StA-47 L	38	Scotland	Adult	–	1
StA-61R	50	Scotland	Adult	F	1
StA-61 L	54	Scotland	Adult	F	1
StA-75R	63	Scotland	Adult	–	1
StA-75 L	3	Scotland	Adult	–	1
Bal-501R	7	Scotland	Adult	–	2
Bal-501 L	12	Scotland	Adult	–	2
Bal-599R	16	Scotland	Adult	–	2
Bal-599 L	22	Scotland	Adult	–	2
Bal-623R	43	Scotland	Adult	–	1
Bal-623 L	51	Scotland	Adult	–	1
Td-43R	13	Italy	Adult	M	1
Td-43 L	18	Italy	Adult	M	1
Td-44R	23	Italy	Adult	M	1
Td-44 L	27	Italy	Adult	M	1
Td-45R	31	Italy	Adult	M	1
Td-45 L	36	Italy	Adult	M	1
Td-46R	39	Italy	Adult	M	1
Td-46 L	44	Italy	Adult	M	1
Td-104R	57	Italy	Adult	M	1
Td-104 L	6	Italy	Adult	M	1
Td-135R	11	Italy	Adult	M	1
Td-135 L	20	Italy	Adult	M	1
Td-271R	28	Italy	Adult	M	1
Td-271 L	33	Italy	Adult	M	1
Cath-3/4R	46	Ibiza	Adult	–	1
Cath-3/4 L	49	Ibiza	Adult	–	1
Cath-295R	60	Ibiza	Adult	–	1
Cath-368/369R	2	Ibiza	Adult	–	1
Cath-368/369 L	5	Ibiza	Adult	–	1
Cath-5 /UE 2115R	10	Ibiza	Adult	–	2
Cath-148/149R	15	Ibiza	Adult	–	1
Cath-148/149 L	21	Ibiza	Adult	–	1
Cath-7/UE 2115 L	24	Ibiza	Adult	–	1
Cath-1/2R	29	Ibiza	Adult	–	1
Cath-1/2 L	32	Ibiza	Adult	–	1

^a^Completeness: 1 = 100–75 %, 2 = 74.9–50 %, 3 = 49.9–25 %, 4 = 24.9–0 %

### Scanning protocols

CT scans of G1 were taken at the Clinical Research Imaging Centre, University of Edinburgh, using a Toshiba Aquilion ONE 320 Detector Row Computed Tomography system, a multidetector CT scanning system. Data were collected using a slice thickness of 0.5 mm and a matrix of 512 × 512 pixels. CT scans of G2 were taken at the Can Misses Hospital, Ibiza, using a GE Medical System HiSpeed NX/I Computed Tomography Scanner. Data were collected using a slice thickness of 1.5 mm and a matrix of 512 × 512 pixels. All data were saved as a Digital Imaging and Communications in Medicine (DICOM) format.

Three-dimensional surface scans of the humeri in G3 were made using a two camera system, each 1.9 megapixels, with a ScanProbe Standard structured light scanner at the University of Bologna. After the initial data acquisition and aligning phases, the point cloud models were input into XOR2 software to generate the final models and saved as stereolithography (.stl) files.

All scan data were randomized before the pair-matching process was tested to minimize bias.

### Segmentation

After randomization, the 31 humeri of G1 and G2 were segmented using AMIRA 5.3.3 to create three-dimensional models using a slightly modified version of Spoor et al.’s Half Maximum Height Value (Online Resource 1) [24]. The 14 humeri from G3 were already three-dimensional surface models and therefore did not need to be segmented. All of the three-dimensional models were then converted from their stereolithography [.stl] format to wavefront [.obj] files.

### Mesh-to-mesh value comparison method — manual

**Fig. 1:**
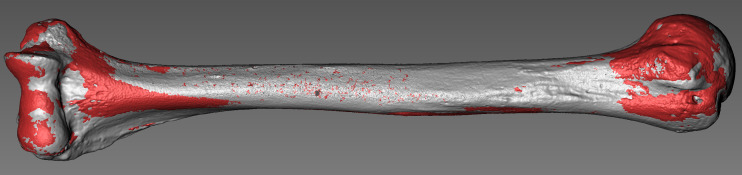
Fine alignment feature. The “fine alignment” feature is used to align and compare the left and right (mirror-imaged) humeri to produce a mesh-to-mesh value. Humerus 20 (Td-135L) is pictured in *red*, while humerus 11 (Td-135R) is pictured in *gray*. The dappling of the *red* and *gray* on the midshaft visually indicates a good match of the two scans, in addition to the obvious size and morphological similarities, while the mesh-to-mesh value confirms it. Image Credit: Mara Karell (color figure online)

The manual MVC method utilized LMI Technologies’ Flexscan 3D to compare all the humeri. The protocol for comparison is as follows:

All right humeri were mirror-imaged using the free software NetFabb basic. All mirror-imaged humeri were then loaded into the Flexscan3D software. One left humerus at a time was then also loaded into the software for comparison against all of the mirrored humeri. All 22 left humeri were subsequently compared to all 23 mirrored-right humeri. To compare any two humeri, both scans were roughly lined up on top of each other using the mouse. Then, the “fine alignment” feature was used in order to obtain a mesh-to-mesh value, which was recorded for comparison (see Fig. 1 for an example).

The mesh-to-mesh values were used as a proxy for pair-matching, where the lowest agreed-upon value indicated the best match. The side of the humeri was initially used to narrow down these values, as a left humerus could not be pair-matched with another left humerus. For the actual test of pair-matching, the three lowest mesh-to-mesh values of each humerus were cross-compared and values were only considered as true matches if both the left and right sides agreed. This was done in order to avoid confusion, for example, if Left Humerus A indicated that it matched best with Right Humerus B, but Right Humerus B indicated that it matched best with Left Humerus C. The standard deviations of mesh-to-mesh values from true pair-matches were calculated to inform a possible cutoff threshold for positive pair matches. In total, comparing all of the humeri to obtain mesh-to-mesh values and recording said values took approximately 45 user-active hours.

### Mesh-to-mesh value comparison method — automated

The automated MVC method utilized Viewbox 4 for comparison. The following settings were used, comparing all meshes to each other from a single folder. The estimated overlap for the scans was 100 %, while the initial position for rough alignment was set at 20. The rough alignment used the nearest neighbor search “Approximate (fast)” with a point sampling of 1 %. It matched point to point, with one hundred iterations. The fine alignment used the nearest neighbor search “Exact with normal compatibility” with a point sampling of 100 %. It matched point to plane, with one hundred iterations. The program then automatically generated an Excel spreadsheet of all of the mesh-to-mesh values for analysis. It should be noted that as this program cannot yet handle comparing 3D surface scans to full CT scan data, the 31 CT scan models were internally hollowed before comparison.

Again, the mesh-to-mesh values were used as a proxy for pair-matching, where the lowest agreed-upon value indicated the best match. The side of the humeri was initially used to narrow down these values. For the actual test of pair-matching, the three lowest mesh-to-mesh values of each humerus were cross-compared, and values were only considered as true matches if both the left and right sides agreed. The standard deviations of mesh-to-mesh values from true pair-matches were calculated to inform a possible cutoff threshold for positive pair matches. The process took approximately five minutes to set up and then approximately 45 hours to run. Only the first five minutes of set up required any activity from the user.

### Comparison of the methods

To compare the efficacy of the two MVC methods, specificity and sensitivity were calculated using Microsoft Excel 2007 [25]. For this study, the gold standard method for comparison was the known humeri pairs. In other words, the main question for assessment was: is this pair of humeri a correct match?

Three previous, high quality studies on visual pair-matching, osteometric comparison, and geometric morphometric pair-matching were also analyzed using sensitivity and specificity in order to directly compare the results of the two MVC methods.

## Results

For the manual version of MVC, the sensitivity and specificity were 100 % and 100 %, respectively (Table 2). Additionally, the type of scan data did not seem to affect the accuracy of the manual MVC version. For the automated version of MVC, the sensitivity and specificity were 95 % and 60 %, respectively (Table 3). This was because, although the automated version correctly identified all of the true negatives in the sample, it mistakenly paired Td-46L (44) with StA-75R (63) and indicated that StA-75L (3) and Td-46R (39) did not have pair-matches present; in other words, that StA-75 L (3) and Td-46R (39) were negatives.

**Table 2:** Sensitivity and specificity results for the manual mesh-to-mesh value comparison method

True positives	42
False negatives	0
False positives	0
True negatives	3
Sensitivity	100 %
Specificity	100 %

**Table 3:** Sensitivity and specificity results for the automated mesh-to-mesh value comparison method

True positives	38
False negatives	2
False positives	2
True negatives	3
Sensitivity	95 %
Specificity	60 %

The mesh-to-mesh values of both versions were analyzed to see if a single threshold value could be used to match pairs instead of the entire Excel sheet matrix. For the manual MVC method, the average mesh-to-mesh value of all of the true matches was 0.638 mm and the standard deviation was 0.176 mm. True match values ranged from 0.402 to 1.225 mm. Two standard deviations from the mean value of 0.638 mm accurately captured all but one of the true match mesh-to-mesh values present. However, attempting to use this value of 1.035 mm as a cutoff for determining pair-matches did not work, as it included an additional 16 values which were not true matches. Similarly, for the automated MVC method, the average mesh-to-mesh value of all the true matches was 1.07 mm and the standard deviation was 0.310 mm. True match values ranged from 0.524 to 1.84 mm. Two standard deviations captured all but one of the true match mesh-to-mesh values. However, attempting to use this value of 1.68 mm as a cutoff for determining pair-matches did not work, as it included 51 additional values which were not true matches. This suggests that utilizing the selection method of a matrix, where both the right and left sides have to agree on the lowest mesh-to-mesh value match, is a better selection method than a single threshold value, at least for humeri. For an example of a mesh-to-mesh Excel matrix and the associated calculations, see Online Resource 2.

The results of comparing Adams and Konigsberg’s visual pair-matching study [5], Garrido-Varas and colleagues’ geometric morphometric pair-matching study [4], and Byrd and Adams’ study on osteometric comparison [6] to the manual and automated MVC methods are found in Table 4. It should be noted that all three studies analyzed different bones and that in the case of Byrd and Adams’ study [6], the authors were attempting to associate different bones, such as a humerus to a radius, not just pair-match the same bone. Similarly, as Garrido-Varas and colleague’s study cannot detect *negatives* (i.e., non-pair matches), the specificity cannot be calculated and is effectively 0 % [4].

**Table 4:** Sensitivity and specificity results comparing all pair-matching methods

Method	Authors	Sensitivity	Specificity
Visual pair-matching^a^	Adams and Konigsberg [7]	91 %	100 %
Osteometric comparison^a^	Byrd and Adams [8]	42 %	96 %
Geometric morphometrics	Garrido-Varas et al. [6]	100 %	Not possible
Manual MVC	This study	100 %	100 %
Automated MVC	This study	95 %	60 %

^a^Calculations can be found in Online Resources 3 and 4

## Discussion

Ideally, especially in regards to courtroom admissibility and returning more remains to their communities, new tools for distinguishing commingled remain should be highly accurate with known error rates, as determined by statistical certainty. In this study, a new method utilizing digital three-dimensional models of bone was tested to assess its impact on pair-matching skeletal elements accurately. The MVC method was invented as a simple attempt to compare the entire surface of two three-dimensional objects and obtain a single value which accurately expresses the similarities between the homologous points of three-dimensional geometry represented. Utilizing the entire geometry of a bone for comparison was expected to improve the results of individualization over the existing methods which use only two-dimensional or partial three-dimensional measurements. Two different versions of the method, one manual and the other automated, were assessed using sensitivity and specificity calculations.

The results obtained from this study have significant implications in light of the demand in forensic anthropology for methods that are more objective and reliable, with known error rates [12]. Foremost, this study uses calculations of specificity and sensitivity to objectively assess the two different versions of the MVC method, in turn creating known error rates for each. Moreover, the manual MVC method seems to outperform the established techniques of visual pair-matching, osteometric comparison, and geometric morphometrics in terms of reliability of pair-matching, with the added benefit of actually having known error rates, unlike visual pair-matching. As the MVC method does not require biological landmarking, it also reduces error across different practitioners. Additionally, another advantage of the method is that it is sex-, population-, and chronology-independent. This drastically simplifies the work of forensic experts when dealing with unclear sets of commingled remains, which can potentially be of different populations or from different time periods. Finally, if the MVC pair-matching method could be applied to mass disasters, it could significantly reduce the cost and burden of DNA testing every bone or bone fragment.

As for a hypothesis regarding why the automated version of MVC is less accurate than the manual version, there are several possible factors. First, though the Flexscan 3D software is based on ICP, from brief experimentation with the software, it also uses additional algorithms to prioritize which surfaces are matched together. Given that the automated MVC method failed to correctly match complete specimens of two different scan types and groups (G1 and G2), the lack of these unknown additional algorithms seems plausible. These algorithms could simply be those for prioritization as mentioned before, or could be other, unknown ones. As the software and its algorithms are proprietary, the exact algorithms may never be known. Second, though different settings for Viewbox, such as point-to-point comparison versus point-to-plane comparison and all of the different iterations of rough and fine alignment, have been tested, other settings, such as different percentages of overlap for cases of fragmentation [26], still need to be investigated. Thus far, the results do not seem to be overly sensitive to the settings, beyond the expected drastic decrease in accuracy of 1 versus 100 % point sampling during the fine alignment stage, but this may change as all of the possibilities of the software are fully explored.

It must also be noted that though the MVC method was tested using two commercial software programs, there is the possibility that the method works just as well on other, free software programs. LMI Technologies, for example, have just released K-Scan, a free program which seems to be identical to Flexscan 3D. Similarly, other common modeling programs such as Mesh Lab or CloudCompare may also work.

Most of the bones tested in this study were complete and in good taphonomic condition, with no visible markers of pathology. This means that the extent and range of the effects of taphonomy, pathology, and fragmentation must be tested for how they impact both the manual and automated MVC method in the future, before it can be applied in the field.

Additionally, scan quality is of the utmost importance when using a method which relies on the accuracy of said scans. Any issues which change the surface quality of a scan, such as improper hole filling from 3D surface scans or CT scan slices that are too thick, will negatively affect the comparison process. Similarly, in our brief experimentation so far, it appears that taphonomy and fragmentation can affect scan quality and thus comparison. Therefore, more research into the extent of these effects must be done before guidelines for scan quality can to be set in order to ensure consistency of results.

**Fig. 2:**
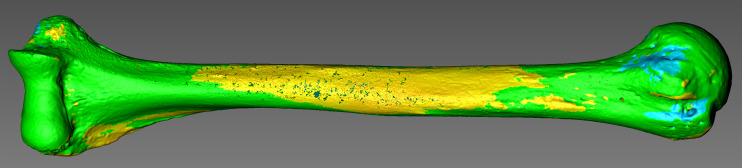
Deviation analysis feature. Example of the deviation analysis feature, where colors indicate regions of size differences. The *green* indicates the areas where the two humeri being compared differ by less than 0.645 mm, the *yellow* where the comparison humerus is 0.654 mm bigger than the reference humerus, and the *blue* where the comparison humerus is 0.654 mm smaller than the reference humerus. Notice how these areas of difference correspond to major muscle attachment features, such as the deltoid tuberosity. Image Credit: Mara Karell (color figure online)

Furthermore, the deviation analysis feature in the Flexscan3D software could be explored to help quantify and catalogue different types and prevalence rates of bilateral asymmetry in addition to the MVC method. Once a mesh-to-mesh value has been created, the deviation analysis feature color-codes the regions of distance between two mesh (such as all of the locations where mesh A is 0.6 mm larger than mesh B are blue, etc.) and allows one to visually inspect similarity. Previous studies on the feature have included measuring the erosion of dinosaur footprints [27]. See Fig. 2 for an example of the deviation analysis feature.

Digital three-dimensional models of bone are permanent, portable, and allow for remote analysis of remains. Though three-dimensional modeling technologies can be expensive, there are a variety of options that range in price, meaning that portable three-dimensional scanners are potentially a field usable tool [20]. As technology progresses and hardware becomes less expensive, it is likely that digital three-dimensional models of bones and techniques that utilize these models will be used more and more routinely. Finally, although the MVC method was developed to pair-match human skeletal elements, it can potentially be used as a means of comparison of any two objects with identical or symmetrical components.

## Conclusion

This study tested two different versions of the novel mesh-to-mesh value comparison method, one manual and the other automated, for accuracy of pair-matching humeri. Both versions were assessed using sensitivity and specificity calculations. The most effective method was the manual MVC method, utilizing the LMI Technologies Flexscan3D software, which had a sensitivity of 100 % and a specificity of 100 %. The automated MVC method, utilizing the Viewbox software, had a sensitivity of 95 % and a specificity of 60 %. These values place the manual version of the MVC method among the most accurate methods available for pair-matching skeletal elements. There is further research to be done to improve both versions of the method including testing the effects of taphonomy, pathology, and fragmentation on the process, as well as expanding the method to other paired bones, reducing the overall cost, and fine-tuning the automated comparison algorithms. This study has demonstrated, however, that the mesh-to-mesh value comparison method is a valuable additional tool for distinguishing commingled human remains.

## Electronic supplementary material

Below is the link to the electronic supplementary material.

Online Resource 1 Segmentation. (PDF 132 kb)

Online Resource 2 Example of Mesh-to-mesh Matrix Calculation. (PDF 192 kb)

Online Resource 3 Sensitivity and Specificity Calculations for Visual Pair-Matching. (PDF 6 kb)

Online Resource 4 Sensitivity and Specificity Calculations for Osteometric Comparison. (PDF 6 kb)
