# Supplementary material for: A novel method for pair-matching using three-dimensional digital models of bone: mesh-to-mesh value comparison
Source: Int J Legal Med. 2016 Mar 10;130(5):1315–22. doi: 10.1007/s00414-016-1334-3 (PMC4976056; doi:10.1007/s00414-016-1334-3)
Supplement: Supplementary file 1 [file 414_2016_1334_MOESM1_ESM.pdf]

Segmentation

Though the segmentation process used for this study followed the protocol set forth by Spoor et al. [1], slight changes were made to the process in order to increase the consistency of randomization. Therefore, the exact protocol followed and the reasoning for the changes in the protocol are below.

Initially, the program ImageJ was used to calculate the HMHV, by calculating the average grey value of every third image of the CT scans. However, when these threshold values were applied, they often did not properly select the trabecular bone of the proximal and distal ends of the humeri. This is not surprising because the HMHV, as Coleman and Colbert note [3], applies a single average threshold value to a data set of hundreds of slices and therefore may not accurately portray all morphological structures. Furthermore, Coleman and Colbert [3] also note that selecting a threshold value can be complicated due to the different levels, or exposure, of different CT scanners and CT scan viewers because the same materials appear to be different grey values.

Histogram Adjustments

To solve these problems, first, the level (exposure) histogram in AMIRA was manually adjusted to create a true black value based on the highest amount of the lowest grey value and a true white value based on the maximum grey value present for each scan (see Figures 1 and 2 below for example of these histograms). This step ensured that all of the scans were now 'exposed' in the same way and thus all of the material present could be viewed in the same manner by the naked eye. Second, the HMHV from ImageJ was slightly manually adjusted using the threshold histogram in AMIRA to more accurately reflect the true contours of bone. Though Coleman and Colbert [3] caution manually adjusting the threshold value, because quantitative

histogram values were used, the adjustments can be consistently recreated and are consistent values. To insure this consistency, all of the level and threshold histogram values were graphed after having been manually adjusted. The graph confirmed that the value creation was indeed consistent and showed an average threshold value of -689.

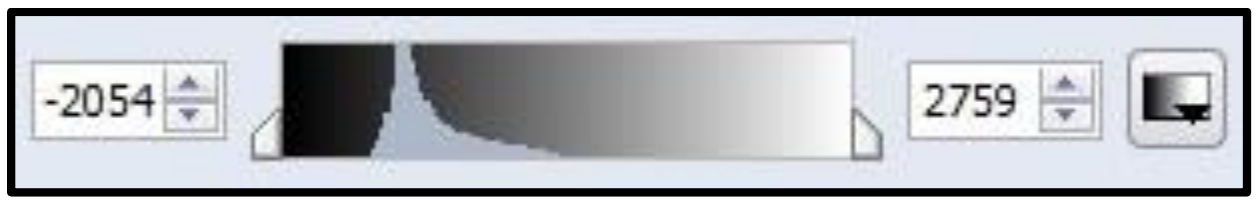

Figure 1 – Level (grey value) histogram before adjustment

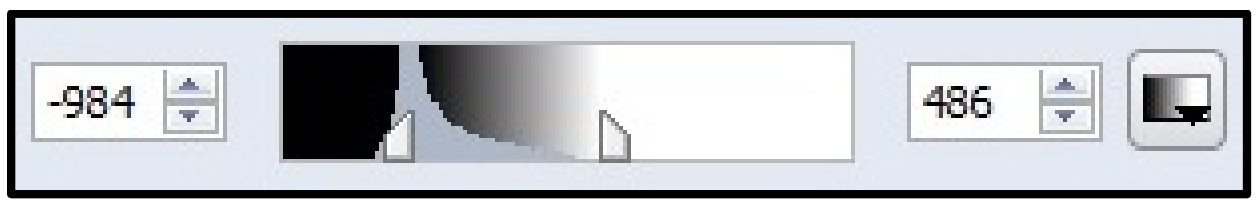

Figure 2 – Level (grey value) histogram after adjustment. Notice how the left slider is at the highest grey value peak and the right slider is at the maximum grey value present

Selecting Segments

Using the aforementioned threshold values, the 'magic wand' tool in AMIRA was used to go through the cross-sectional slices of each humeri and select the appropriate bone portion. The 'magic wand' tool works by selecting contiguous pixels of the user-specified grey value range. Though the 'thresholding' feature could have been used to automatically select every value within the proper threshold range mentioned before, the 'magic wand' tool was chosen instead so as to be able to exclude the table pieces that were also included in the scans and fell within the threshold density range for bone. When all of the slices had been selected, the selected parts, now termed 'Labels' in AMIRA, were transferred back to the main object pool viewer. In the main object pool viewer, the 'surface gen' feature was use to merge these selected parts together to create a cohesive polygonal surface mesh, with constrained smoothing. These polygonal surface meshes were then saved as Stereolithography (.stl) files with Little Endian compression.

References

1. Spoor CF, Zonneveld FW, Macho GA (1993) Linear measurements of cortical bone and dental enamel by computed tomography: applications and problems. Am J Phys Anthropol 91: 469-484.
2. Hounsfield GN (1973) Computerized transverse axial scanning (tomography). 1. Description of system. Br J Radiol 46: 1016-1022.
3. Coleman MN, Colbert MW (2007) Technical note: CT thresholding protocols for taking measurements on three-dimensional models. Am J Phys Anthropol 133: 723-725.
4. Fajardo RJ, Ryan TM, Kappelman J (2002) Assessing the accuracy of high-resolution X-ray computed tomography of primate trabecular bone by comparisons with histological sections. Am J Phys Anthropol 118: 1-10.
