# Supplementary material for: A novel method for pair-matching using three-dimensional digital models of bone: mesh-to-mesh value comparison
Source: Int J Legal Med. 2016 Mar 10;130(5):1315–22. doi: 10.1007/s00414-016-1334-3 (PMC4976056; doi:10.1007/s00414-016-1334-3)
Supplement: Supplementary file 2 [file 414_2016_1334_MOESM2_ESM.pdf]

Mesh-to-mesh Values Example Data

Left sides	Right sides -->				
↓	1	2	7	8	10
3	2.002	2.511	3.06	2.011	1.654
4	2.296	1.249	1.51	0.546	1.784
5	2.169	0.65	1.87	1.092	2.191
6	2.296	3.207	1.729	3.113	2.194
12	0.716	1.609	0.634	1.781	1.454
14	0.688	2.458	3.007	2.021	2.284

Text in Red = Potential match due to low mesh-to-mesh value (value < 1)

Three lowest values starting with the Lefts (going across each row from left to right)

Lefts	Rights -->				
↓	1	2	7	8	10
3					
4		1.249	1.51	0.546	
5		0.65		1.092	
6					
12	0.716		0.634		1.454
14	0.688				

Lowest Value | Second Lowest Value | Third Lowest Value

Text in Yellow = True Match

Three lowest values starting with the Rights (going down each column from top to bottom)

Lefts ↓	Rights --> 1	2	7	8	10
3					
4		1.249	1.51	0.546	
5		0.65		1.092	
6					
12	0.716		0.634		1.454
14	0.688				

Lowest Value | Second Lowest Value | Third Lowest Value

Text in Yellow = True Match

Combined lowest values

Lefts ↓	Rights --> 1		2		7		8		10	
3										
4			1.249	1.249	1.51	1.51	0.546	0.546		
5			0.65	0.65			1.092	1.092		
6										
9										
12	0.716	0.716			0.634	0.634			1.454	1.454
14	0.688	0.688								

Lowest Value (L) | Lowest Value (R) | True Negative

Text in Yellow = True Match

True Pair Matches

Left	Right
3	63
4	8
5	2
6	57
12	7
14	1
