# Supplementary material for: A novel method for pair-matching using three-dimensional digital models of bone: mesh-to-mesh value comparison
Source: Int J Legal Med. 2016 Mar 10;130(5):1315–22. doi: 10.1007/s00414-016-1334-3 (PMC4976056; doi:10.1007/s00414-016-1334-3)
Supplement: Supplementary file 3 [file 414_2016_1334_MOESM3_ESM.pdf]

Visual Pair-Matching	
True positive	22
False negative	2
False positive	0
True negative	14
Sensitivity	91.67%
Specificity	100.00%
